# Trabecular Titanium for Orthopedic Applications: Balancing Antimicrobial with Osteoconductive Properties by Varying Silver Contents

**DOI:** 10.1021/acsami.2c11139

**Published:** 2022-09-07

**Authors:** Anna Diez-Escudero, Elin Carlsson, Brittmarie Andersson, Josef D. Järhult, Nils P. Hailer

**Affiliations:** † Ortholab, Department of Surgical Sciences—Orthopaedics, Uppsala University, Uppsala 751 85, Sweden; ‡ Zoonosis Science Center, Department of Medical Sciences, Uppsala University, Uppsala 751 85, Sweden

**Keywords:** additive manufacturing, titanium, silver coating, osseointegration, antibacterial

## Abstract

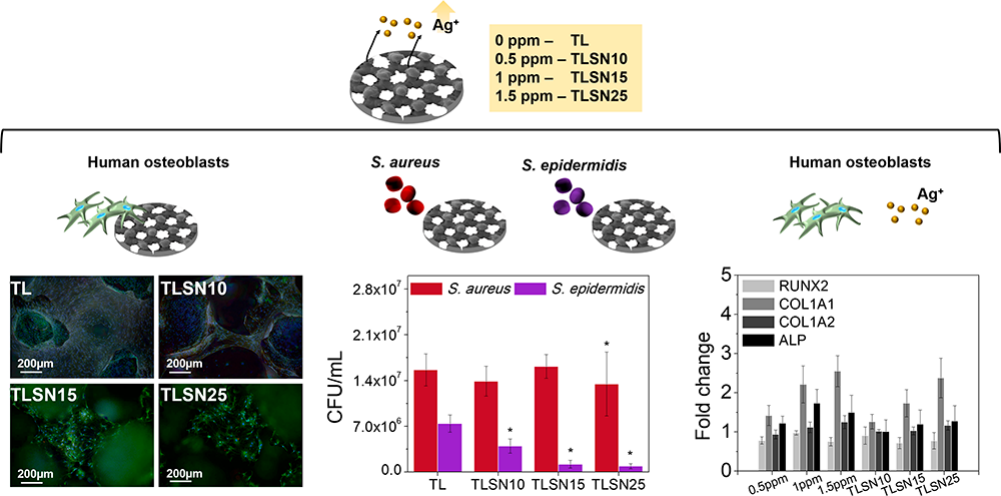

Periprosthetic joint infection (PJI) and implant loosening are the most common complications after joint replacement surgery. Due to their increased surface area, additively manufactured porous metallic implants provide optimal osseointegration but they are also highly susceptible to bacterial colonization. Antibacterial surface coatings of porous metals that do not inhibit osseointegration are therefore highly desirable. The potential of silver coatings on arthroplasty implants to inhibit PJI has been demonstrated, but the optimal silver content and release kinetics have not yet been defined. A tight control over the silver deposition coatings can help overcome bacterial infections while reducing cytotoxicity to human cells. In this regard, porous titanium sputtered with silver and titanium nitride with increasing silver contents enabled controlling the antibacterial effect against common PJI pathogens while maintaining the metabolic activity of human primary cells. Electron beam melting additively manufactured titanium alloys, coated with increasing silver contents, were physico-chemically characterized and investigated for effects against common PJI pathogens. Silver contents from 7 at % to 18 at % of silver were effective in reducing bacterial growth and biofilm formation. *Staphylococcus epidermidis* was more susceptible to silver ions than *Staphylococcus aureus*. Importantly, all silver-coated titanium scaffolds supported primary human osteoblasts proliferation, differentiation, and mineralization up to 28 days. A slight reduction of cell metabolic activity was observed at earlier time points, but no detrimental effects were found at the end of the culture period. Silver release from the silver-coated scaffolds also had no measurable effects on primary osteoblast gene expression since similar expression of genes related to osteogenesis was observed regardless the presence of silver. The investigated silver-coated porous titanium scaffolds may thus enhance osseointegration while reducing the risk of biofilm formation by the most common clinically encountered pathogens.

## 1 Introduction

Total joint arthroplasty (TJA) is a common surgical procedure with more than 10 decades of proven success and effectiveness in restoring limb function and mobility.^1^ TJA entails the partial or total substitution of damaged joints and bone by a prosthetic material, usually a metallic implant, to treat diseases such as osteoarthritis (OA), rheumatoid arthritis, paediatric hip diseases, and hip fractures.^2^ OA is described as a disease of cartilage including also damage to the subchondral bone, synovium, and adjacent adipose tissue,^3^ with a multifactorial etiology. The main risk factors are increasing age and female sex, with genetics and obesity also playing major roles, together with biomechanical factors. The increase in life expectancy, aging population, sedentary lifestyles, and obesity epidemic are some of the main causes of the rise of cases of OA.^4^ To exemplify this scenario, more than a million arthroplasties are performed annually, and the projections over the next 20 years estimate an increase of approximately 400% for total knee arthroplasties (TKA) and a 284% increase for total hip arthroplasties (THA) in the USA.^5^

Despite the great historical success of TJA procedures, periprosthetic joint infection (PJI) is the main early cause of post-operative complications and prosthetic failure^6,7^ and implies costly treatments consisting of prolonged systemic antibiotic administration and multiple revision surgeries that negatively impact patients’ life.^8^ Given the increasing incidence of OA and TJA, the incidence of PJI has also increased,^9^ and robust strategies to overcome PJI are highly needed. PJI rates are up to 1.2% upon implantation and can increase to 4.6% in revision surgery cases.^10−12^ PJI occurs by the bacterial colonization of the implant surface, later developing a biofilm, which resists surgical debridement and antibiotic treatments.^8^ The resistance of a mature biofilm at later stages of PJI necessitates revision surgery where implants are exchanged, and this can be performed in the setting of either one- or two-stage procedures. Although the cure rates after such revision procedures range from 72% to 95% after treatment with antibiotics, temporary implantation of antibiotic-loaded polymethyl methacrylate (PMMA) spacers, and implantation of new prostheses, a frustratingly high proportion of failures is observed.^11,13^ Several challenges exist in the diagnosis of the causative organism of PJI to offer adequate treatments, and still the development of antibiotic resistant bacteria prevents these patients from yielding full recovery.

In this context, surface modifications of the implants inserted during revision surgery can help reducing bacterial colonization and hence avoid the relapse of PJI. Increasing efforts have been made toward developing new strategies to provide antibacterial surfaces on implants, although few have successfully been applied into clinics. Experimental approaches, yet far from clinical application, mostly rely on sputtering techniques including different metallic ions such as copper, zinc, zirconium, or strontium incorporated as coatings.^14−17^ The most common clinical strategies are based on the use of drug-eluting materials: PMMA, which is well established and used either for spacers or for permanent fixation of definitive implants, and, more experimental, hydrogels such as hyaluronic acid and polylactic acid that can be loaded with single or dual antibiotics to treat PJI during revision surgery.^18,19^ Furthermore, novel metal ion-loaded PMMA, such as silver-loaded PMMA, has shown promising results to treat PJI.^20^ In fact, silver, known for its antibacterial properties through several cellular pathways, has offered promising results as antibacterial coating. Clinically, silver-coated arthroplasty components have demonstrated efficacy in preventing PJI,^21^ especially when applied in high-risk patients in orthopedic oncology. Noteworthy, recent clinical trials also include the use of bacteriophages^22^ or antimicrobial peptides,^23^ which augur to offer alternatives to treat PJI.

When performing a TJA, the implant is anchored in the surrounding bone. Two main fixation principles exist, direct fixation of metal intended for bone ingrowth into host tissue or cemented fixation of components by the use of PMMA. Uncemented implants are mostly manufactured from titanium-based alloys that undergo surface modifications such as grit blasting to increase osteointegration. Thus, when using uncemented implants, biocompatibility and osteointegration of prosthetics are crucial for TJA success, and aseptic loosening is the other major cause of implant failure apart from PJI. When developing novel implants intended for uncemented fixation that also provide antibacterial properties, it is paramount that antimicrobial surface modifications do not interfere with bone formation and, ultimately, bone ingrowth. Importantly, the race for the surface, as coined by Gristina referring to the competition established between bacteria and cells to colonize the implant surface,^24^ i.e., between host tissue and nosocomial strains, will dictate the success of the TJA. In particular, with regard to silver-coated prostheses, the release of silver can detrimentally affect mammalian cells and cause damage to surrounding fibrous and bone tissues; thus, a balance between antibacterial effects and biocompatibility of silver needs to be identified.

Another major issue associated with metallic implants is the mismatch between the bone and implant elastic modulus, which can potentially lead to stress shielding, resulting in massive bone resorption, which will render a poor bone to implant contact and possible dislocations. For this reason, porous metals such as titanium and tantalum have been proposed^25−27^ and further promoted thanks to the development of additive manufacturing (AM). AM enables customization of implants to adapt to patient needs, the defect location, and biomechanical demands of the implant site. In fact, porous metals, in addition to provide enhanced anchoring to bone tissue, have been demonstrated to promote osteogenesis and osteointegration by their porous architectures.^28,29^ Noteworthy, the higher surface area of porous metals can render them more susceptible to bacterial colonization, increasing the risk of PJI. For this reason, antibacterial coatings on porous metals that enhance osteocompatibility while simultaneously reducing the risk of PJI are sought after.

Recently, we demonstrated that the balance between antibacterial and osteogenic properties can be controlled by a physical vapor deposition process that regulates the overall silver content, not only on dense materials but also on porous trabecular metals. The support of cell proliferation and bactericidal effects of thinner silver coatings was effective on dense materials, but larger silver amounts, while antibacterial, impaired primary cells survival.^30^ However, the extrapolation of the same silver content coating on porous substrates exhibited suboptimal antimicrobial effects, especially against *Staphylococcus aureus* (*S. aureus*).^31^ The increased surface area available together with micrometer features at the scaffolds surface, which is known to drive bacterial colonization,^32^ was ascribed to the reduced antibacterial effects.

Therefore, in this work, we investigated silver-coated porous titanium alloys (Ti6Al4V) produced by additive manufacturing with increasing silver content coatings. The porous structure aims at improving osteointegration by resembling the porous trabecular structure of the bone, which was evaluated *in vitro* with primary human osteoblasts. The antibacterial effects of silver-coated porous titanium with three different concentrations of silver were evaluated against the two most common causative agents of PJI,^33−35^*S. aureus* and *Staphylococcus epidermidis* (*S. epidermidis*), both derived from actual PJI patients. The balance between antibacterial and osteogenic effects was assessed by culturing human osteoblasts or PJI-derived *Staphylococci* on the different titanium samples. Furthermore, the silver ion release from the silver-coated titanium scaffolds was measured, and the direct effects of silver ions on human osteoblasts were analyzed. Our objective was thus to identify the optimal balance between antimicrobial effects on the one hand while not disturbing osteogenic potential on the other hand.

## 2 Experimental Methods

### 2.1 Samples Production and Characterization

Samples and surface coatings were produced by Waldemar LINK GmbH (Germany). Trabecular porous scaffolds consisting of 12.5 mm diameter and 2 mm height discs were additively manufactured from extra low interstitial Ti6Al4V alloy powder (ELI Ti6Al4V) through an electron beam melting technique (Arcam EBM Q10 Plus) as previously reported.^31^ The manufactured discs (Trabeculink: TL), with a 1 mm solid base and a 1 mm trabecular top structure, were subsequently coated with silver by arc physical vapor deposition (PVD, Voestalpine eifeler alpha 400P) using pure silver and titanium targets under a nitrogen atmosphere, after glow discharge cleaning (Trabeculink Silver Nitride: TLSN). The PVD deposition was established with varying cathode silver targets (up to 12) and varying deposition times, depending on the final silver amount desired.

The scaffolds were morphologically assessed through field emission scanning electron microscopy (FE-SEM, Merlin Zeiss, Germany) and white light interferometry (WLI, NexView Zygo, Ametek Inc., Weiterstadt, Germany) using a scanned area of 167 × 167 μm^2^ and 50× magnification. The cross-sectional morphology was further investigated using a focus ion beam (FIB, Zeiss Crossbeam 550). A layer of platinum was deposited on each scaffold surface (2 kV, 2 nA for 10 min) over an area of 30 × 30 μm^2^. Before milling, a local protective layer of 0.6 μm platinum was deposited on the cross-section area. A coarse milling using gallium ions with a current between 65 and 30 nA was used to perform a cross-section of 25 μm width and 20 μm depth. Afterward, a finer milling and polishing of the cross-section was achieved by reducing the current from 7 nA to 300 pA. The cross-sections were imaged using energy-selective backscattered detector (ESB). Measurements on the coating thicknesses were obtained from the cross-sections of three independent samples using ImageJ software and corrected by the projection angle used during FIB imaging (54°).

The surface chemical composition of the scaffolds was investigated semiquantitatively by elemental analysis using energy-dispersive X-ray spectroscopy (EDS, Merlin Zeiss, Germany) at an operating voltage of 15 kV and a working distance of 8.5 mm. Additional quantitative analysis was performed by X-ray photoelectron spectroscopy (XPS, Physical Electronics Quantera II, USA, Inc.) using an Al monochromatic Kα beam, at 25 W and 15 kV. The spectra were acquired using 20 ms time per step and 55 eV as pass energy. The bulk composition of the scaffolds was characterized by X-ray diffraction (XRD, D8 Advance, Bruker, Germany), at 40 kV and 40 mA, using Bragg–Brentano geometry, a Cu Kα anode, from 20° to 80° over a 2θ range. The collected data was compared to experimental patterns for titanium (Ti, JCDPS 44-1294), titanium nitride (TiN, JCPDS 38-1420), and silver (Ag, JCPDS 01-1164).

### 2.2 Cell Cultures with Primary Human Osteoblasts

The human osteoblasts (OB) were isolated from human femoral heads after assessment by the Swedish Ethical Review Authority (approval number: 2020-04462) following previously published protocols.^36^ The obtained bone parts were finely cut into 1–2 mm fragments, rinsed with phosphate-buffered saline (PBS, Gibco) and cell culture media (alpha modified minimum essential medium, α-MEM, Merck, KGaA, Darmstadt, Germany), and placed for expansion in 25 cm^2^ flasks supplemented with complete media (CM) containing α-MEM, 10% fetal bovine serum (FBS, Merck, KGaA, Darmstadt, Germany), 1% penicillin/streptomycin, and 0.5% amphotericin. After 4 weeks, the cells were transferred to 75 cm^2^ flasks and further expanded until passage 3 to 6 for the cell culture experiments.

Cell proliferation, differentiation, and mineralization of OB seeded on the scaffolds were assessed at 1, 7, 14, and 28 days. The scaffolds were incubated overnight with complete media(CM) before seeding (preincubation). In the next day, cell culture media were removed, and 10^6^ human OB were seeded in a 50 μL droplet per scaffold. Afterward, the seeded scaffolds were placed in an incubator for 1 h. Finally, 950 μL of complete media were added, yielding a final cell density of 5·10^4^ cells/mL. Control samples consisting of empty wells without scaffolds were included for quantification and cover glass slips for imaging. For the control samples, 950 μL of complete media were directly added after seeding to avoid drying in the incubator. Cell media were refreshed every 2 days along the full 28 days of the cell culture studies. After 7 days of cell culture, osteoinductive media (OIM) were supplemented to the cells. OIM consisted of 10 mM beta-glycerophosphate, 100 nM dexamethasone, and 80 μM ascorbic acid (Merck, KGaA, Darmstadt, Germany) supplemented to the complete media.

A lactate dehydrogenase enzymatic assay (LDH, TOX7, Merck, KGaA, Darmstadt, Germany) was used to quantify cell adhesion and proliferation at 1, 7, 14, and 28 days. At each time point, the scaffolds with cells were rinsed with PBS (×1), transferred to new wells, and lysed with 400 μL lysis buffer (CellLytic, Sigma-Aldrich, Sweden) for 15 min at 300 rpm on a shaker. Following manufacturer’s protocol, 50 μL of cell lysate were mixed with TOX7 reagents, and the absorbance was measured at 690–490 nm in a spectrophotometer (Multiscan Ascent, ThermoFisher Scientific Inc., Waltham, MA). The optical density values were normalized to control samples at each corresponding time and the total area, either the scaffolds area or the well area for control samples. Alkaline phosphatase (ALP) was quantified in cell lysates using an ALP substrate (Merck, KGaA, Darmstadt, Germany) at the corresponding time points (1, 7, 14, and 28 days) to monitor cell differentiation. Following manufacturer’s protocol, 50 μL of cell lysate were mixed with the ALP substrate and the absorbance at 405 nm was measured. The absorbance values were converted to concentration by a standard calibration using *p*-nitrophenol dilutions (Sigma-Aldrich, Sweden) ranging from 0 mM to 1 mM. ALP concentration was normalized to the total protein content in the same cell lysates measured by a bicinchoninic acid assay (BCA, Pierce BCA Protein Assay Kit, ThermoScientific, Rockford, IL) at 540 nm and further normalized to control samples at each corresponding time point. Three technical replicas were used per scaffold type and time point, and the experiments were repeated twice using two different patient-derived OB (TLSN15 and TLSN25) and compared to previous data (TL and TLSN10) consisting of three technical replicas and three biological replicas.

Alizarin red (AR, Merck, KGaA, Darmstadt, Germany) staining was used to quantify the mineral deposits produced by OB on the scaffolds at 14 and 28 days. At each time point, the scaffolds were rinsed with PBS (×1) and fixed with 70% ice cold ethanol for 1 h. Afterward, the scaffolds were rinsed with distilled water and stained with 40 mM AR at pH 4.2 for 10 min at room temperature. Subsequently, the dye was removed, and the scaffolds were rinsed with distilled water (×5), followed by PBS (×1) rinsing for 15 min at 300 rpm. Finally, the AR dye was removed from the scaffolds using 10% wt cetylpyridinium chloride (CPC, Merck, KGaA, Darmstadt, Germany) in 10 mM sodium phosphate solution for 20 min at 300 rpm at room temperature, and the absorbance of the supernatant was quantified in a microplate reader at 562 nm. The absorbance values were expressed as concentration using a standard calibration curve of AR ranging from 0 mM to 0.8 mM. Two technical replicas were used per scaffold type and time point, and the experiment was repeated twice using two independent patient-derived OB. One additional sample per scaffold type without cells was used as a control, stained, and quantified. The values of control samples were subtracted as background from the corresponding scaffolds.

Immunocytochemistry (ICC) was used to visualize cell morphology and the expression of osteocalcin (OCN) at 14 and 28 days on the scaffolds. At each time point, the scaffolds were rinsed with PBS (×1), subsequently fixed in 4% v/v paraformaldehyde at room temperature for 20 min, rinsed with PBS (×3), and permeabilized using 0.1% Triton X-100 (Merck, KGaA, Darmstadt, Germany). The cell cytosol was stained with carboxyfluorescein diacetate (500 nM, 400 μL CFDA, Merck, KGaA, Darmstadt, Germany) for 15 min, followed by normal 10% goat serum (s-1000, Sigma-Aldrich, Sweden) blocking solution in PBS containing 2% BSA and 0.3% Triton X-100 for 30 min. A solution of an OCN antibody (20 μg/mL human/rat OCN, MAB1419, R&D Systems, United Kingdom) in PBS/2% BSA/0.3% Triton X-100 was used to detect OCN expression. The OCN antibody was incubated on the scaffolds overnight at 4 °C. After rinsing with PBS/1% Triton X-100 (×4), the secondary antibody (1:200, goat antimouse, Biotin Novus NB7537, United Kingdom) was incubated for 30 min under agitation at room temperature. Finally, the scaffolds were rinsed with PBS/1% Triton X-100 (×4) and stained with Dylight red (20 μg/mL, ThermoFisher Scientific Inc., Waltham, MA) and DAPI (300 nM, 4'-6-diamidino-2-phenylindole, Invitrogen, Massachusetts, USA), dissolved in PBS for 30 min at room temperature, followed by rinsing with PBS/1% Triton X-100 (×4). The cells on the scaffolds were imaged using a Leica microscope (Leica Dmi8, Mycrosystems CMS GmbH, Wetzlar, Germany). One sample per scaffold and per time point was analyzed.

#### 2.2.1 Silver Release Quantification

Inductively coupled plasma optical emission spectroscopy (ICP-OES, Avio 200, Perkin Elmer, USA) was used to analyze the silver release from silver-coated scaffolds TLSN10, TLSN15, and TLSN25 in human OB cell culture supernatants over 28 days. The cell culture supernatants were collected at 0 (preincubation), 1, 2, 3, 5, 7, 9, 14, 19, 21, 26, and 28 days from triplicate samples, diluted in 2% nitric acid solution (HNO_3_, Merck, Germany), and analyzed with an argon flow (8 L/min) and a pump rate of 1 mL/min. Cell culture media were used as blank controls. The silver concentration values were obtained after using silver standards ranging from 0.01 ppm to 10 ppm and presented as mean ± standard deviation.

#### 2.2.2 Silver Effects on Primary Human Osteoblasts

To study the effects of silver on primary human OB, silver nitrate (AgNO_3_, Sigma-Aldrich) solutions matching the concentrations of the silver release for each silver-coated scaffolds (ICP measurements section 2.2.1) were prepared and supplemented to human OB cell cultures seeded on tissue culture polystyrene (in the absence of the scaffolds) over 28 days. Primary human OB were isolated as described in section 2.2. Cells at passage 3 were seeded in 24-well plates (35.000 cells/mL), and after 24 h, the culture medium was exchanged for treatment media consisting of culture medium supplemented with AgNO_3_ at 0.5, 1.0, or 1.5 ppm, or one of three varying concentrations, which simulated the silver release from the scaffolds (TLSN10, TLSN15, and TLSN25) at each specific time point as quantified by ICP (Table 1). The concentrations simulating those of the scaffolds were named as TLSN simulated (TLSN10s, TLSN15s, and TLSN25s). Control samples consisting of untreated cells were included. Treatment media were refreshed every 2 days. After 7 days of cell culture (6 days of treatment), osteoinductive media (OIM) were supplemented to the cells, i.e., treatment media were thereafter OIM supplemented with AgNO_3_ as above. Cell morphology and health were examined at 3, 14, and 28 days using live-image microscopy (Leica DMi8 Microscope with INCUBATORi8 environmental chamber). Representative optical microscopic images were taken at each corresponding time point (10× magnification, exposure time 12 ms).

**Table 1:** Silver Ion Concentration Profiles for Constant Silver Concentrations and the Simulated Silver Concentrations Simulating the Release of the Silver-Coated Scaffolds^a^

		silver concentration (ppm)						
time (days)	base medium	control	0.5	1.0	1.5	TLSN10s	TLSN15s	TLSN25s
0	CM	0.0	0.5	1.0	1.5	0.7	0.9	0.7
2	CM	0.0	0.5	1.0	1.5	0.7	0.9	1.0
4	CM	0.0	0.5	1.0	1.5	0.2	0.9	1.2
6	OIM	0.0	0.5	1.0	1.5	0.2	1.1	1.3
8	OIM	0.0	0.5	1.0	1.5	0.2	1.1	1.4
10	OIM	0.0	0.5	1.0	1.5	0.1	1.0	1.3
12	OIM	0.0	0.5	1.0	1.5	0.1	1.0	1.3
14	OIM	0.0	0.5	1.0	1.5	0.1	0.8	1.2
16	OIM	0.0	0.5	1.0	1.5	0.1	0.8	1.2
18	OIM	0.0	0.5	1.0	1.5	0.1	0.5	1.2
20	OIM	0.0	0.5	1.0	1.5	0.1	0.4	0.8
22	OIM	0.0	0.5	1.0	1.5	0.0	0.4	0.5
24	OIM	0.0	0.5	1.0	1.5	0.0	0.4	0.5
26	OIM	0.0	0.5	1.0	1.5	0.0	0.3	0.4

a CM: complete media, OIM: osteoinductive media.

Osteogenic-related genes in human OB exposed to silver were analyzed by real-time quantitative polymerase chain reaction (RT-qPCR) at the end of the culture period, 28 days. Human OB were lysed at the end point using 400 μL TRIzol (Invitrogen, Massachusetts, USA) and stored at −20 °C until used. Following the manufacturer’s protocol, ribonucleic acid (RNA) was extracted, and the total RNA yield was quantified using a nanodrop (ND-1000 Spectrophotometer, Thermofisher Scientific Inc., Waltham, MA). Reverse transcription (high-capacity RNA to c-DNA kit, ThermoFisher Scientific Inc., Waltham, MA) was used to transform RNA into complementary DNA (cDNA), and the osteogenic-related genes listed in Table 2 (Thermofisher Scientific, Taqman) were measured by RT-qPCR (7500 Fast RT PCR System, Applied Biosystems, Thermofisher Scientific Inc., Waltham, MA). Collagen type I alpha 1 and 2 (COL1A1, COL1A2), Runt-related transcription factor 2 (Runx2), and alkaline phosphatase (ALP) gene expression were quantified using the Livak’s method, expressed as 2^ΔΔ^CT, and GAPDH was used as a house-keeping gene. Triplicates for each condition were used, and three independent patients’ cells were used.

**Table 2:** Taqman Probes for the Primers Used in Gene Expression Quantification

acronym	name	assay number ID
GAPDH	glyceraldehyde-3-phosphate dehydrogenase	NM_002046.3
Runx2	runt-related transcription factor 2	Hs00231692_m1
COL1A1	collagen type I alpha 1	Hs00164004_m1
COL1A2	collagen type I alpha 2	Hs01028970_m1
ALPL	alkaline phosphatase	Hs01029144_m1

### 2.3 Antimicrobial Effects

The antimicrobial effects of silver coating were evaluated using two patient-derived bacterial strains, *S. aureus* and *S. epidermidis*, obtained from patients treated for PJI at Uppsala University Hospital.

The biofilm formation for each particular strain at 6, 15, 24, and 72 h was directly evaluated using crystal violet (CV) quantification, following previously described protocols.^37,38^ Briefly, a 1 μL loop was used to collect bacterial colonies, which were diluted 1:50,000 in 0.9 % wt sterile sodium chloride (NaCl, 1/50,000 dilution, Sigma-Aldrich, Sweden), followed by a 1:10 dilution in tryptic soy broth (TSB, Soybean-Casein Digest Medium, BD Bioscience, Sweden). Afterward, 1 mL of TSB solution containing bacteria (approx. 10^3^ CFU/mL) was placed on each scaffold in a 24-well plate and incubated aerobically at 37 °C. At each respective time point, the bacteria solution was removed, and the scaffolds were rinsed with NaCl (×1), followed by fixation using 99.5% methanol (Sigma-Aldrich, Sweden) at −20 °C for 30 min. After removal of the methanol solution, the scaffolds were left to dry and stained with 0.1% crystal violet solution for 45 min. Afterward, the dye was removed and the scaffolds were carefully rinsed with PBS (×3). Finally, the scaffolds were decolorized with 600 μL of 96% ethanol (Sigma-Aldrich, Sweden) for 20 min in a shaker, and the supernatants were measured at 595 nm in a transparent 96-well plate. Three replicas per scaffold type were used for CV quantification. One scaffold of each was used as a control, incubated in TSB without bacteria, and stained with CV. The absorbance values of control scaffolds were subtracted from the bacteria-containing scaffolds for quantification.

Additionally, biofilm formation after 72 h was indirectly evaluated by counting colony forming units (CFU) after enzymatic detachment as previously reported.^31,38^ A total of 3 mL of bacterial solution (approx. 10^3^ CFU/mL, prepared as for CV measurements) in TSB was placed on the scaffolds inside a 50 mL falcon tube and incubated at 37 °C for 72 h. Afterward, the bacteria solution was removed, and the scaffolds were rinsed with NaCl (×2). An enzymatic solution containing 2 mg/mL dispase and 4 mg/mL collagenase was placed on the rinsed scaffolds (600 μL), incubated for 2 h at 37 °C at 220 rpm. Finally, the scaffolds were vortexed for 2 min, and the enzymatic solution was diluted and plated in blood agar plates for CFU counting after 24 h of incubation at 37 °C. Three replicas per scaffold type were used, and the experiment was repeated three times.

### 2.4 Statistics

Statistical analyses were performed using SPSS IBM software. Homogeneity of variance was assessed using Levene’s test, differences between groups were evaluated by one-way ANOVA and Tukey’s post-hoc test in case of equal variances, and Games-Howell test when homogeneity of variances was not present. The significance level was defined as *p* < 0.05. The physichochemical characterization was performed using duplicates for XPS, triplicates for EDS quantifications, and triplicates for WLI with five measurements per sample. The biological characterization is presented as a mean ± standard error from triplicate scaffolds and duplicate biological replicas using independent patients’ cells. CV staining and quantification are presented as a mean ± standard error from triplicate samples. CFU counts are presented as box plots including mean (box middle line), median (squares), and confidence intervals (90%).

## 3 Results and Discussion

### 3.1 Scaffolds Characterization

**Figure 1:**
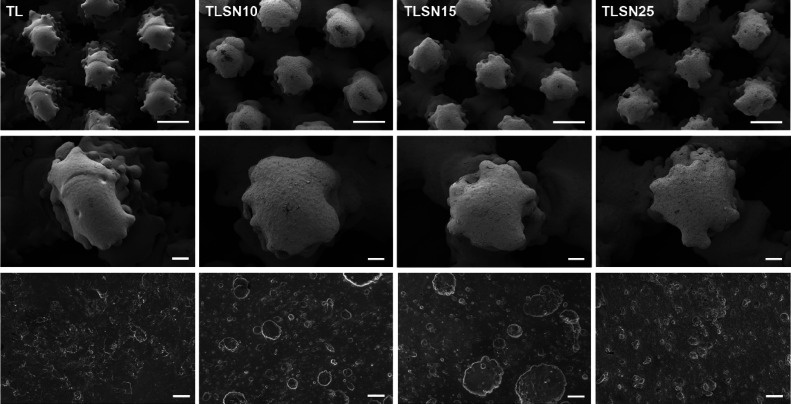
Scanning electron images of the four scaffolds illustrating their porous trabecular structures (first row, scale bar: 500 μm), the morphology of the 3D-printed pillars (second row, scale bar: 100 μm), and detailed surface morphology depicting silver islands from SN-coated scaffolds (third row, scale bar: 5 μm).

The morphology assessment by SEM of the four different scaffolds depicted their porous structures consisting of pores with sizes of 500–600 μm (Figure 1). All scaffolds showed the trabecular 3D-printed structures, with no changes in their surfaces. The overall porosity and pore size have been shown to be crucial in cell proliferation and differentiation processes.^39^ Similar additively manufactured metallic implants with pore sizes ranging from 500 μm to 650 μm improved cell proliferation^40^ and osteoblast maturation.^41^ In addition to the effects on cell behavior, porous metallic scaffolds can potentially improve the implant anchoring to host tissue (bone to implant contact surface), while reducing the stress shielding effects.^25^ At higher magnifications, the porous Ti structure of the control containing the lowest amount of silver (TLSN10) depicted a more homogeneous surface, while scaffolds with higher silver contents (TLSN15 and TLSN25) presented slightly more numerous silver islands (Figure 1, third row). The islands became smaller as the Ag content increased, similarly to previous reported work using PVD and pure dense titanium substrates.^42,43^

**Figure 2:**
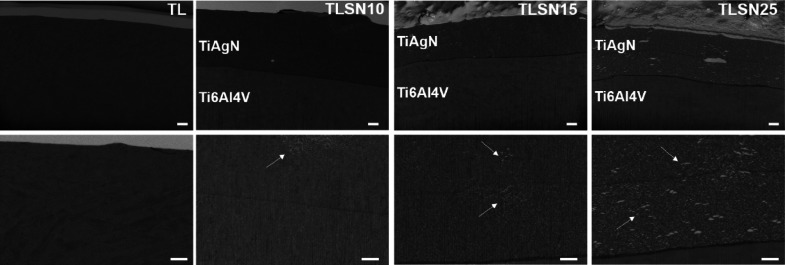
Cross-section-backscattered electron images after FIB treatment of TL, TLSN10, TLSN15, and TLSN25, illustrating the different layers of the scaffolds consisting of the Ti6Al4V substrate, and the TiAgN coating (first row; scale bar: 1 μm). Higher magnification images (second row; scale bar: 500 nm) depicting the microstructure of TL and the TiAgN coatings in TLSN10, TLSN15, and TLSN25.

Titanium-based alloys such as Ti6Al4V are known to have poor wear resistance, and several ceramic coatings are applied to reduce the release of metallic ions to surrounding tissue by improving wear and corrosion resistance.^44^ A common ceramic hard coating with decades of success in uncemented prostheses is titanium nitride (TiN), typically applied with a thickness between 1 and 4 μm,^45^ and which endows implants with improved hardness, wear, and corrosion resistance, while ensuring good biocompatibility.^45,46^ The PVD coating applied in the present work using titanium (Ti) and silver (Ag) targets allows incorporating silver as an antimicrobial agent with a tight control of the silver content by adjusting the amount of silver targets and resulting in hard and thick coatings of titanium silver nitride (TiAgN). The cross-sectional images illustrated the varying thicknesses and the TiAgN coating and subjacent Ti6Al4V microstructures (Figure 2). The TL control subjacent microstructure consisted of both acicular and equiaxial grains, compatible with similar additively manufactured Ti-alloys,^47,48^ and depicting a mix of equiaxed and acicular grains from alpha/beta stabilizers typical for the alloying elements, aluminum and vanadium, respectively. Coated TLSNs depicted a major equiaxed grain microstructure for the Ti6Al4V substrate (Figure 2, first row). The coating thicknesses varied from TLSN10 to the higher Ag contents in TLSN15 and TLSN25. TLSN resulted in the thickest coating with an average thickness of 10.7 ± 1.2 μm, while TLSN15 and TLSN25 depicted more similar thicknesses, 6.5 ± 0.3 μm for TLSN15 and 7.5 ± 0.3 μm for TLSN25. At higher magnification (Figure 2, second row, white arrows), white Ag inclusions increased as the Ag content in the coating increased, with TLSN25 showing the largest silver inclusions.

**Figure 3:**
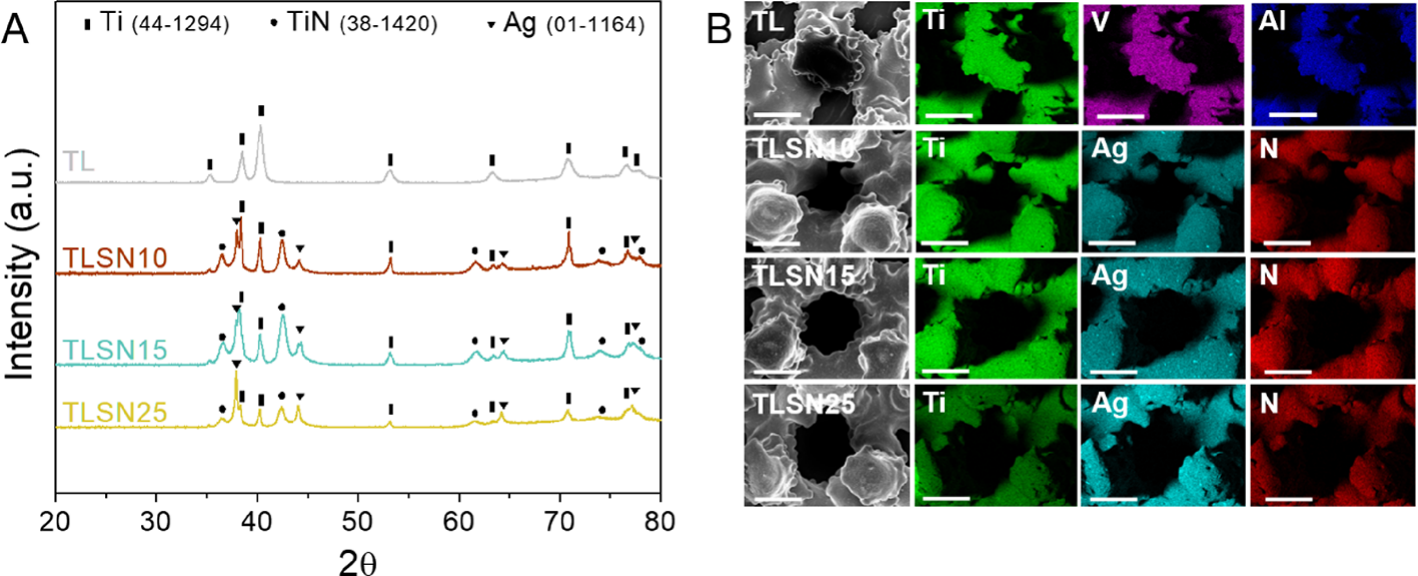
X-ray diffractograms of the uncoated scaffolds (TL) and silver-coated scaffolds with increasing silver contents (A). Energy-dispersive X-ray spectroscopy (EDS) elemental mapping of the scaffolds illustrating the main elemental compositions (B): green for titanium, purple for vanadium, dark blue for aluminum, cyan for silver, and red for nitrogen (scale bar: 250 μm).

To confirm the presence of silver and the chemical cues of each scaffold, both bulk and surface analyses were performed. Crystallographic phase analysis of the scaffolds (Figure 3A) exhibited mainly the titanium (Ti) phase in all four scaffolds. Additional peaks corresponding to silver (Ag) and titanium nitride (TiN) were found in silver-coated scaffolds. The peak intensity for Ag (37.934°, peak index (1 1 1)) increased from TLSN10 to TLSN25. At the lowest Ag content in TLSN10, two distinguishable peaks corresponding to Ti and Ag phases were observed, while as the Ag increased (TLSN15 and TLSN25), the two peaks overlapped.

At the surface level, as investigated by EDS, the Ag atomic percentages (Table 3) were found to be 13.8 at % for TLSN15 and 17.7 at % for TLSN25, compared to control TLSN10 with 7 at % Ag, as previously reported.^31^ Colored elemental mapping representative for each scaffold (Figure 3B) showed the homogenous distribution of silver over the scaffolds’ surface, and the presence of the Ag islands observed by SEM (Figure 1, third row) was shown by higher intensity Ag (cyan points).

**Table 3:** Atomic Elemental Semiquantification by EDS and Surface Compositional Atomic Ratios Ag/Ti and N/Ti Quantified by XPS

	EDS mapping analysis						XPS atomic ratio	
sample	Ti (at %)	Al (at %)	V (at %)	O (at %)	N (at %)	Ag (at %)	Ag/Ti	N/Ti
TL	64.7	8.3	3.7	16.3				
TLSN10	33.2				56.5	7	0.2	1.0
TLSN15	19.0				60.9	13.8	0.3	1.4
TLSN25	17.6				58	17.7	0.6	1.4

**Figure 4:**
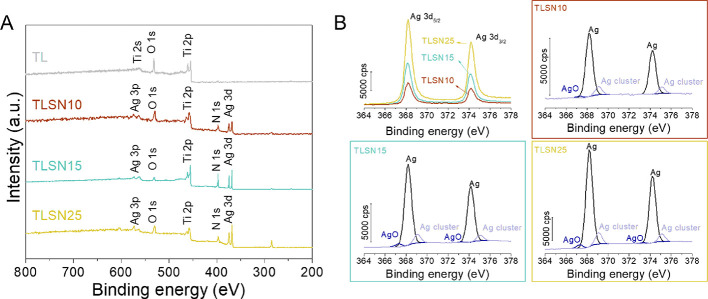
XPS survey spectra of the different scaffolds, uncoated TL and silver-coated TLSN10, TLSN15, and TLSN25 (A), and deconvoluted XPS data for silver Ag 3d on silver-coated scaffolds TLSN10, TLSN15, and TLSN25 (B).

To further support the semiquantitative values of the Ag content acquired by EDS, XPS was used to quantify the silver content at the surface level and to investigate the silver oxidation state (Figure 4). High-resolution survey spectra showed the main elements consisting of titanium and oxygen (Ti, O) for control TL and Ti, O, together with Ag and N for the silver-coated scaffolds (Figure 4A). The calculated atomic ratios for Ag/Ti and N/Ti ascribed to the PVD coating process showed an increase as the Ag content on the coating increased (Table 3). This was further evidenced by the intensity counts for the Ag 3d detailed spectra (Figure 4B). Additionally, the oxidation state of the silver composing the coatings revealed a major metallic silver component (Figure 4B) regardless of the total silver amount. This was consistent with the nitrogen atmosphere used for the PVD coating process, which prevented silver oxidation. Regardless of the silver content, due to the absence of oxygen during the PVD process, the oxidation state of the silver present in the coating remained similar between the three coated scaffolds. Control coating TLSN10 depicted 88.4 ± 1.4 at % metallic silver (368.3 eV), while 86.5 ± 8.7 at % was found for TLSN15 and 87.5 ± 1.5 at % for TLSN25. Two other states were identified in all silver-coated scaffolds compatible with silver clusters and silver(II) oxide. Silver clusters were identified at higher binding energies (369.2 eV) and accounting for 9.4 ± 0.0 at % for TLSN10, 11.0 ± 1.1 at % for TLSN15, and 10.0 ± 0.0 at % for TLSN25. Similar coatings achieved through PVD reported similar nanometric silver clusters.^49^ Finally, discrete amounts of silver(II) oxide were found at lower binding energies (367.4 eV), accounting for 2.2 ± 1.5 at % for TLSN10, 2.5 ± 0.2 at % for TLSN15, and 0.8 ± 0.7 at % for TLSN25.

Surface roughness is known to play key roles in cell attachment and behavior. Several strategies consisting of nanotextures or reducing surface roughness to nanoscales have been proved successful in reducing initial bacterial colonization.^50,51^ White light interferometry at the printed struts and pillars was used to characterize the roughness and kurtosis of the scaffolds (Table 4). The average surface roughness of the scaffolds was approx. 5 μm, without statistically significant differences between them. Slight variations of the surface kurtosis were observed, especially for higher silver contents in TLSN15 and TLSN25 scaffolds, compatible with the silver islands observed by SEM (Figure 1) and by FIB cross-sectioning (Figure 2, white arrows) which increased the height distribution of the surface profiles.

**Table 4:** Surface Characteristics of the Scaffolds Measured by WLI Depicting Surface Roughness and Kurtosis Values

	surface roughness	
sample	*R*_a_ (μm)	*R*_ku_ (μm)
TL	4.7 ± 1.2	1.7 ± 0.2
TLSN10	5.0 ± 1.4	1.9 ± 0.7
TLSN15	5.0 ± 1.1	2.0 ± 0.9
TLSN25	4.7 ± 1.0	2.2 ± 0.7

### 3.2 Osteoblast Cell Cultures

**Figure 5:**
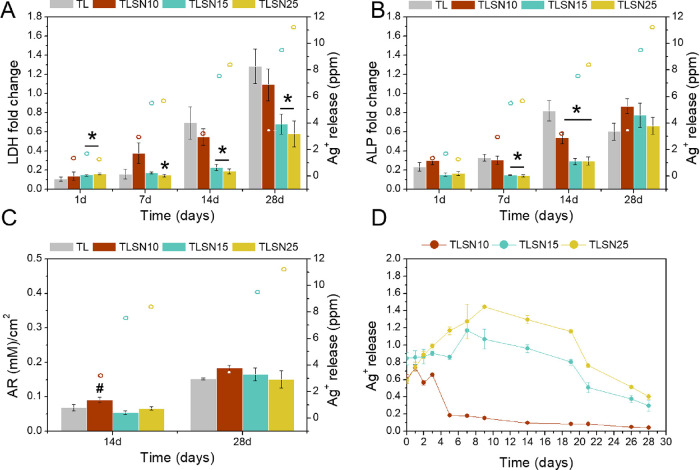
Human OB proliferation (A), differentiation (B), and mineralization(C) over a 28 day culture period, analyzed by LDH, ALP, and AR, respectively. Silver levels at each corresponding time point are included for comparison in each single graph (hollow dots). Silver release from the silver-coated scaffolds into cell culture supernatants analyzed through ICP over the 28 days culture period (D). * denotes statistical significance (*p* < 0.05 Tukey post-hoc) between samples at each time point, and # states significant differences (*p* < 0.05 Games–Howell post-hoc) between samples.

Cell colonization, proliferation, and differentiation are crucial to achieve successful long-term stability of uncemented titanium implants. These phenomena often dictate whether the implant osteointegrates into the host bone or whether fibrous encapsulation occurs. The assessment of scaffolds direct cytocompatibility with eukaryotic cells becomes even more relevant when silver is present. Despite the use of silver as an antibacterial agent, silver is also known to impair mammalian cells viability. Silver ion levels of 2.5 ppm were cytotoxic to mesenchymal stem cells,^52^ and levels of 1 and 1.5 ppm impaired human monocytes and T-cells viability, respectively.^53^ The proliferation of human OB on the four investigated porous scaffolds (Figure 5A) indicated slightly higher proliferation rates on non-coated TL scaffolds, although all silver-coated scaffolds also promoted cell proliferation. At 1 day, significantly higher cell amounts were found on higher silver content scaffolds TLSN15 and TLSN25. Previous studies have shown that cell adhesion can improve due to surface roughness, although mostly ascribed to nanometer ranges of roughness.^54^ Despite the similar surface roughness values in all investigated scaffolds, differences in the height profiles could explain the higher adhesion at day 1 observed for TSLN15 and TLSN25, which showed higher surface kurtosis. Importantly, a recent study in similar additively manufactured scaffolds also pointed at higher cell adhesion rates on rougher 3D-printed Ti6Al4V compared to a forged analogue,^48^ although lacking macroporosity. All the investigated scaffolds showed regular porous structures ascribed to the lattice applied in the manufacturing process. Thus, effects of the total surface area available to cell adhesion are comparable within all four scaffolds. Nevertheless, the specific surface area derived from the coatings can still play a significant role. After 7 days, the potential topographical effects on cell proliferation clearly vanished, where uncoated TL and lower silver content TLSN10 induced higher cell proliferation. At 7, 14, and 28 days, cell proliferation was significantly reduced for higher silver contents, TLSN15 and TLSN25. Importantly, at these time points, the silver release for TLSN15 and TLSN25 significantly increased compared to TLSN10 (Figure 5A, hollow dots, and Figure 5D). It could be hypothesized that the chemical cues of the scaffolds in terms of silver ion release may surpass the physical features effects on the cell metabolic activity. Similarly, despite lacking signs of cytotoxicity to human OB proliferation, metabolic activity was slightly impaired at 7 and 14 days as the silver content increased on the TLSN15 and TLSN25 samples, as shown by the lower ALP activity (Figure 5B). However, even though slightly lower ALP levels were observed, after 28 days, differences between the four scaffolds were not statistically significant. Thus, despite the fact that silver ions reduced the metabolic activity of human OBs, these attained similar levels of differentiation regardless of the silver content (Figure 5B). Similarly, mineral deposition assessed by AR staining and quantification after 14 days was reduced on high silver content TLSN15 and TLSN25 samples compared to low silver content TLSN10 controls, but no statistically significant differences were observed between uncoated control TL and silver-coated TLSN15 and TLSN25. Nevertheless, AR concentration recovered after 28 days, with no statistically significant differences between the four scaffolds (Figure 5C).

To allow comparison with the previous work, the silver release from the silver-coated scaffolds through 28 days in cell culture media was monitored (Figure 5D). Noteworthy, due to the complexation ability of silver ions with present proteins such as albumin^55^ and the affinity for important biochemical anions such as sulfur and nitrogen present in cells and tissues,^56^ the assessment in cell culture media embodies a more representative environment to assess silver release than non-physiological media such as water. TLSN10, with the lowest silver content, showed a rapid maximum silver release (0.74 ppm at 1 day). As the silver content increased, a delay in the maximum peak of silver release was observed; TLSN15 had its maximum silver concentration released to the cell culture media at 7 days (1.2 ppm), while TLSN25 maximum silver release was identified at 9 days (1.5 ppm). Importantly, the silver release profiles (Figure 5D) showed a decay in ionic silver release for TLSN10 after 3 days, while TLSN15 decay was delayed until 7 days, and TLSN25 silver release decreased only after 9 days. This phenomenon might be due to the silver content in the coating, where TLSN25 with 17.7 at % of silver could sustain a more prolonged silver ionic release. Given the antibacterial effects of silver ions, it is paramount to assess a continuous release of such rather than a burst release, given the different proliferative rates of PJI pathogens. The major presence of metallic silver in all coatings (Figure 4B) might be the key to achieve a continuous release, as seen for higher silver content scaffolds TLSN15 and TLSN25. The major metallic silver state in the coatings will eventually ionize into ionic silver through contact with body fluids and oxygen,^56−58^ granting the biochemical activity of silver as an antibacterial agent for a sustained period of time. In this line, nanoparticulate complexes based in supramolecular fullerene silver nitrate proved efficiency in sustaining silver ion release to reduce bacterial growth, although compromised cell proliferation was also observed.^59^

**Figure 6:**
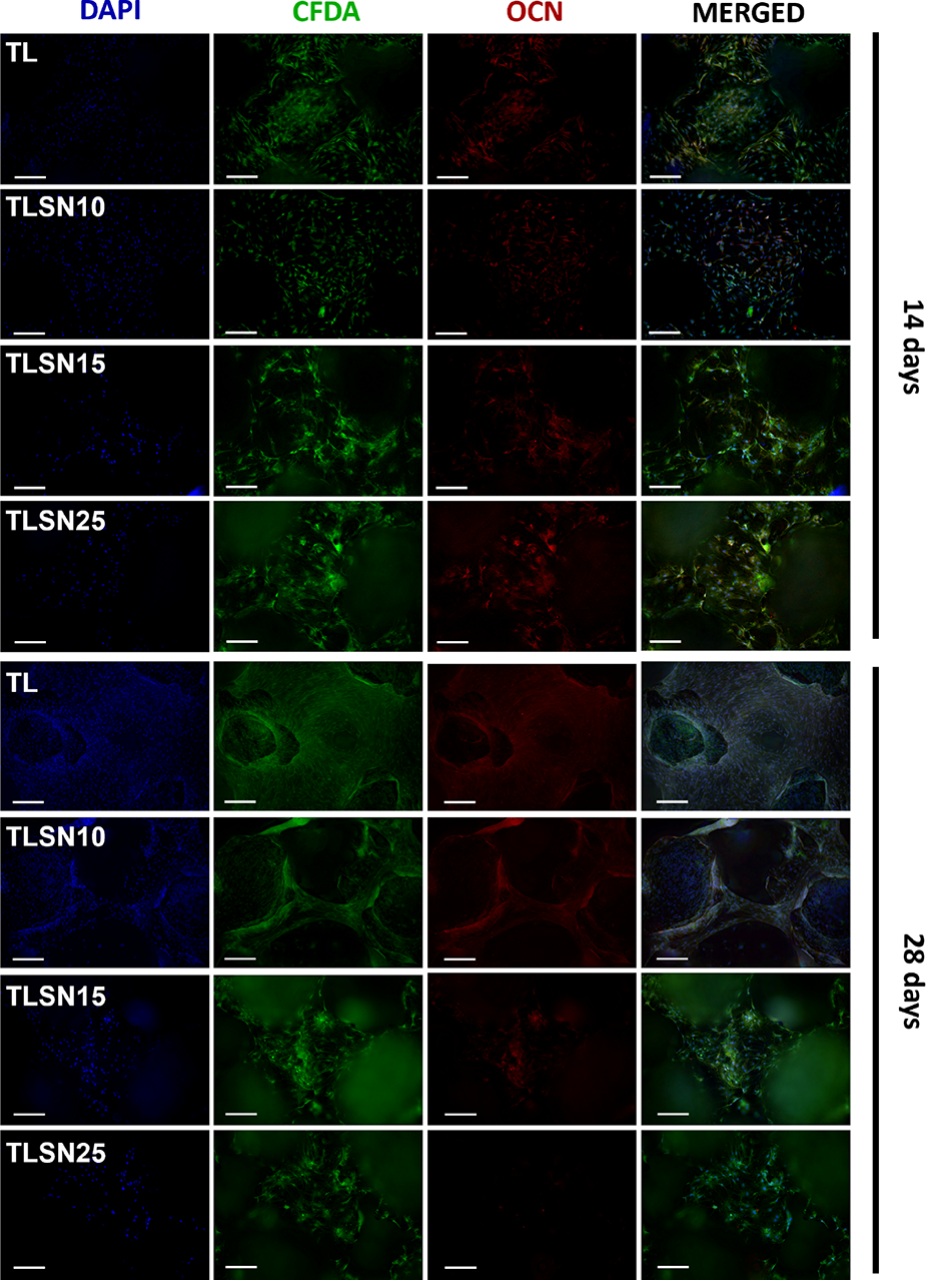
ICC images of human OB at 14 and 28 days observed by fluorescence microscopy illustrating cell nuclei (blue), cell cytoskeleton (green), and osteocalcin (red), imaged after 14 and 28 days; scale bar: 200 μm.

The morphology, matrix deposition, and mineralization of human OB assessed by ICC after 14 and 28 days (Figure 6) correlated well with the proliferation rates, differentiation, and mineralized bone matrix quantified by LDH, ALP, and AR (Figure 5A–C). A lower number of cell nuclei was observed at 14 days compared to 28 days for all scaffolds; a lower number of cell nuclei was observed on TLSN15 and TLSN25 samples with a higher silver content than on lower silver content TLSN10 and on uncoated TL samples. Given the porous nature of the scaffolds and the high surface area for cell colonization, topographical effects showed a higher influence on cell adhesion at 14 days, whereas after 28 days, the chemical environment derived from higher silver content scaffolds might have played a more important role, as shown by a more disrupted cell cytoskeleton (Figure 6, green staining). At 28 days, while a mat of cells was observed on uncoated TL and low silver content TLSN10, both at the printed pillars and at the pore bottom, cell colonization on higher silver TLSN15 and TLSN25 seemed to be restricted to the printed pillars rather than the pores. The higher ionic silver release, which might have accumulated locally, at the pore concavities, could result in a lower cell activity. Additionally, surface grooves and nooks could also be ascribed as cell cytoskeleton inhibitors due to impediment of cell bending and subsequent movement.^60^

The OCN intensity as a measure of the mineralization process, and relevant for osteoblastic maturation, was higher on TLSN15 and TLSN25 at 14 days compared to 28 days, oppositely to the quantification of mineral deposits by AR quantification (Figure 5C), which depicted similar levels between all scaffolds. The ICC images evidenced higher OCN intensity for TLSN10 and TL control samples than on the higher silver content TLSN15 and TLSN25 samples at 28 days. However, it is important to note that a delay between cell markers expression detected by ICC and the calcium deposits appearance quantified by AR might explain the differences between the two observations.

#### 3.2.1 Silver Effects on Human Osteoblasts

**Figure 7:**
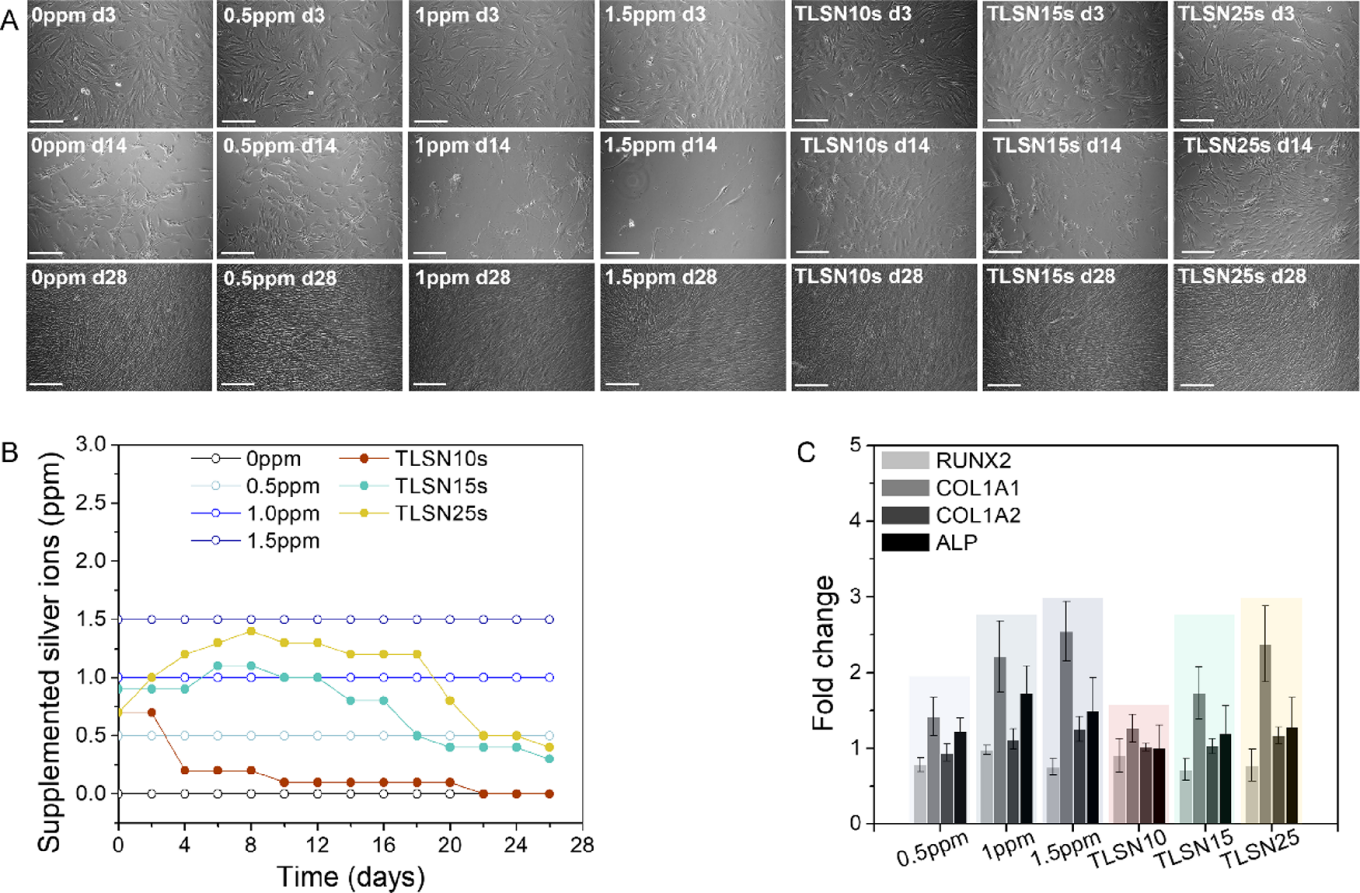
Optical images of human OB, representative from one patient, after 3 (first row), 14 (second row), and 28 days (third row) depicting the morphology and coverage upon silver ions supplementation; scale bar 200 μm (A), according to constant levels of silver or scaffold-matching silver levels (B). The metabolic activity of human OB from three independent patient’s cells assessed by gene expression of osteogenic-related genes at 28 days (C).

Despite the clinical use of silver coating on arthroplasty components, the coating is limited to parts of the implants that have no direct bone contact. The concerns of side effects of such implants related to silver toxicity on mammalian cells have limited extensive applications.^21^ Thus, it is paramount to assess the viability of cells in the presence of silver and, importantly, using relevant models such as the use of primary human cells from different patients to reflect the variability between individuals. To eliminate the effects of the scaffold architecture and surface features and to focus on the effects of silver ions on the human OB morphology and metabolic activity, silver ions were directly added to human OB cultures over 28 days. The cultures were supplemented with constant silver amounts (0.5, 1, and 1.5 ppm) and with silver concentrations simulating the release from the scaffolds quantified by ICP (Figures 5D and 7B). The morphology of human OB at 3, 14, and 28 days was imaged by optical microscopy (Figure 7A). A few differences were observed at 3 days between all samples, either when human OBs were exposed to constant silver levels or to the simulated scaffolds’ release; however, after 14 days of cell culture, a clear reduction in cell coverage was observed, strongly evident for 1 and 1.5 ppm Ag^+^ supplementation. Importantly, the simulated scaffolds’ release (TLSN10s, TLSN15s, and TLSN25s) showed a well-spread cell morphology, with similar cell coverage as that of 3 days. Noteworthy, at the end of the culture period, a full cell coverage was achieved for all conditions, both the steady silver supplementation, and the simulated silver release from the scaffolds. To further characterize the possible differences in the human OB metabolic activity exposed to the different Ag^+^ release profiles, osteogenic-related gene expression was quantified at 28 days (Figure 7C). Overall, the osteogenic related genes showed no variations of gene expression between the different modes of silver exposure, and no statistical differences were found in any gene expression from either of the three different patients. A slight downregulation of RUNX2 was observed for all samples when compared to controls. Similar levels of COL1A2 were observed in all samples, and a slight upregulation of COL1A1 and ALP was detected in all samples after 28 days of culture, but without statistical significance.

### 3.3 Antimicrobial Effects

**Figure 8:**
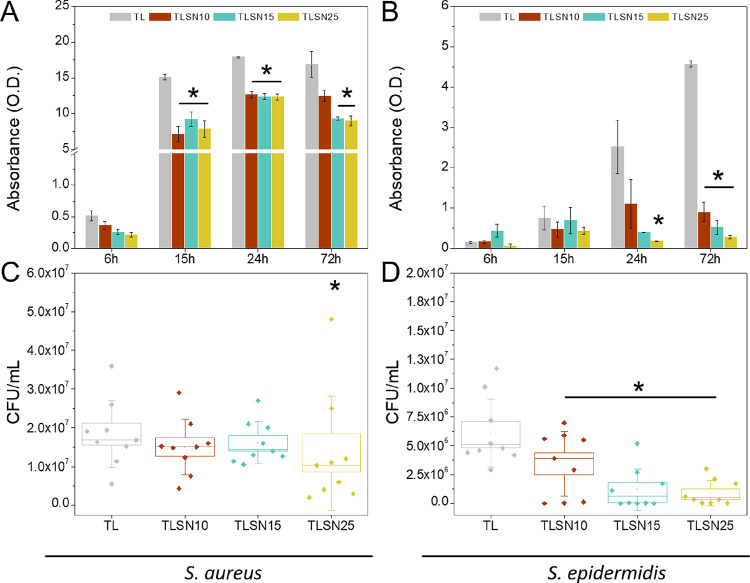
Bacterial growth on the porous scaffolds measured as a kinetic growth using crystal violet at 6, 15, 24, and 72 h for *S. aureus* (A) and *S. epidermidis* (B); indirect biofilm quantification at 72 h after enzymatic detachment and CFU counts for *S. aureus* (C) and *S. epidermidis* (D). * denotes statistical differences (*p* < 0.05 Tukey post-hoc) between samples at each specific time point.

Metal ions have been proposed as alternative strategies for antibacterial applications due to their diverse bactericidal mechanism.^61^ Silver ions are known to disturb cell metabolism by inducing reactive oxygen species (ROS), interacting with thiol and amino groups, therefore impairing bacteria membrane function, damaging lipids, proteins, and DNA.^62^ Importantly, these mechanisms are not exclusive to bacteria but apply also to eukaryotic cells, hence establishing the therapeutic window for silver is crucial to attain functional coatings to sustain and improve osteogenesis while providing antibacterial effects. In a PJI scenario, two gram-positive *Staphylococci* strains are the most concurrent infection pathogens, *S. aureus* and *S. epidermidis*.^34^ The growth kinetics of PJI patient-derived *S. aureus* and *S. epidermidis* were investigated on the four different scaffolds at 6, 15, 24, and 72 h by CV staining and quantification (Figure 8A,B). Clear different growth rates were observed for each strain, similarly to previous studies employing both strains in commercial forms,^63^ which could be related to the antibacterial silver effects between the strains. *S. aureus* growth occurred more rapidly on the scaffolds than *S. epidermidis*, reaching 3–10-fold higher growth rates. The silver coatings significantly impaired the evolution of *S. aureus* (Figure 8A); a significant reduction of *S. aureus* was observed at 15 and 24 h for all silver-coated scaffolds, while only higher silver contents TLSN15 and TLSN25 were able to sustain significant antibacterial effects after 72 h. The growth of *S. epidermidis* on the scaffolds was lower than that of *S. aureus*, and although lower levels were observed for all silver-coated samples, significances were found only for the highest silver coating TLSN25 after 24 h of incubation (Figure 8B). After 72 h, all silver coatings exhibited a significant reduction of *S. epidermidis* growth compared to uncoated TL scaffolds, and a clear reduction trend was observed as the silver content increased in the coating. Previous studies have demonstrated great efficacy of silver to reduce *S. aureus* growth up to 7 days with silver contents of 15 wt % using titanium oxide nanotubes impregnated with silver; similarly, the antibacterial effects were ascribed to the continuous release of silver from the nanotubes by both their higher surface area and their hollow depth.^64^ Currently, several other metallic ions are investigated for their potential antibacterial properties such as copper and zinc in synergy with silver for more versatile antibacterial surface coatings.^17^ A particularly interesting study working with Methicillin resistant bacteria engineered enzyme responsive assemblies capable of targeting Methicillin resistant *S. aureus* and locally releasing silver ions from silver nanoparticles.^65^ Their system allowed a sustained silver ion release with an extreme low biosafety risk (silver ion release in the range of 0.01 ppm over 24 h compared to 0.9 ppm for regular silver nanoparticles ion release). Providing a sustained release of silver ions can be paramount to tackle bacterial growth since the development of PJI entails several sequenced events beyond bacterial colonization, the last one being the development of the biofilm. Bacterial biofilm development on prosthetics can be a devastating complication due to the biofilm resistance to antibiotics or even mechanical debridement.^8,66^ Therefore, biofilm formation after 72 h incubation was also assessed indirectly through CFU counting method to support the direct CV staining and quantification. Previous investigations have shown the importance and variability in biofilm assessment on solid surfaces depending on the methodology used,^38^ and at clinical level, this becomes even more relevant for accurate microorganism identification and successful diagnosis and treatment.^67,68^ On the other hand, the assessment of silver antibacterial properties both in the long term and in the presence of the biofilm have been pointed as crucial to render silver coating approaches clinically relevant. In the case of silver nanoparticles, a high tolerance of wastewater biofilms to silver concentration has been proven.^69^ This phenomenon has also been extended to implant-associated biofilms, where a 1000-fold decrease in susceptibility of biofilm bacteria to antibiotics has been shown.^70^ The biofilm formation on the scaffolds by *S. aureus* (Figure 8C), despite depicting a reduction in the mean values of total CFU/mL in silver-coated scaffolds compared to uncoated, was only significant for the highest silver content scaffolds, TLSN25. *S. epidermidis* indirect biofilm evaluation (Figure 8D) correlated well to the direct kinetic growth in Figure 8B. A trend on reduction of biofilm formation as the silver content increased was observed, and significant differences were obtained. The antibacterial effects observed in the long term might be ascribed to the slow release of silver ions from the pristine metallic silver by the higher content silver contents TLSN15 and TLSN25. This was evident for *S. epidermidis*, where only long-term exposure both direct and indirectly resulted in a significant reduction of bacterial accumulation.

Overall, higher silver contents demonstrated effectiveness in reducing *S. aureus* and *S. epidermidis* growth and biofilm formation. Higher susceptibility to silver was observed for *S. epidermidis* than for *S. aureus*; this is likely related to their distinctly different growth rates, but other species and strain-specific traits may also contribute. In line with previous work on the same PVD-engineered coatings, we have shown that the same coating, on a non-porous lower surface area scaffold, was able to reduce bacteria biofilm significantly;^30^ however, the same coating on porous high surface area additively manufactured scaffolds reduced the antibacterial effects of silver.^31^ Thus, higher contents of silver in the coatings were needed to achieve similar pathogen reduction on porous additively manufactured scaffolds compared to dense substrates. Importantly, the coatings demonstrated no signs of cytotoxicity against primary human osteoblasts and additionally allowed cell proliferation and differentiation. Nevertheless, some effects due to silver ions release on the metabolic activity were observed, which, however, were reduced later in the experiments.

## 4 Conclusions

The establishment of a therapeutic window for silver coatings of prostheses is still unclear, and the issue becomes even more relevant with the development of porous metal implants with a larger surface area. These porous implants are more sensitive to bacterial colonization than non-porous implants, potentially increasing infection rates. The balance between osseointegration-osteogenesis in host tissue, and the presence of antibacterial properties is paramount to reduce infection rates, enhance osseointegration, and reduce loosening, the main modes of failure of joint replacements. Novel trabecular porous Ti alloy-based materials were successfully developed by additive manufacturing, and post-processed to obtain coatings with increasing silver amount, thus enhancing the antibacterial properties of the scaffolds. The silver coatings tested here exhibit bactericidal efficacy against most dominant strains in PJI cases, *S. aureus* and *S. epidermidis*, and antibacterial effects dependent on the silver content. Additionally, a strain-dependent effect was found for similar silver levels, indicating a higher susceptibility of *S. epidermidis*. Importantly, all silver content coatings exhibited cytocompatibility with primary human osteoblasts. Although silver-coated scaffolds reduced the metabolic activity of cells, no impairment in cell differentiation and maturation could be found on silver-coated scaffolds nor was direct exposure to silver concentrations simulating long-term exposures toxic to osteoblasts.
